# Retraction: Health risk assessment of exposure to chlorpyrifos in pregnant women using deterministic and probabilistic approaches

**DOI:** 10.1371/journal.pone.0351612

**Published:** 2026-06-15

**Authors:** The PLOS One Editors

The *PLOS One* Editors retract this article [1] due to concerns about compromised peer review and compliance with PLOS policy on Manipulation of the Publication Process.

ET, MMA, and AF did not agree with the retraction. SSD and IA responded but expressed neither agreement nor disagreement with the editorial decision. RK either did not respond directly or could not be reached.
